# Applying the 5P framework to improve cancer prevention in European health systems

**DOI:** 10.1057/s41271-026-00627-8

**Published:** 2026-05-27

**Authors:** Hamideh Frühwein, Susanne Theis, Marie Neu, Francesca Alt

**Affiliations:** 1 Institute for the History, Philosophy, and Ethics of Medicine, University Medical Center of the Johannes Gutenberg University Mainz, Mainz, Germany; 2 Department of Obstetrics and Gynegology, University Medical Center of the Johannes Gutenberg University Mainz, Mainz, Germany; 3 Childhood Cancer Center, University Medical Center of the Johannes Gutenberg University Mainz, Mainz, Germany

**Keywords:** Cancer Prevention, Policy, Equity, 5P model of healthcare

## Abstract

Cancer remains one of the leading causes of premature mortality in Europe, with nearly half of all cases attributable to modifiable risk factors. While preventive strategies are well-established, their implementation across European health systems remains fragmented, underfunded, and insufficiently aligned with population needs. This paper applies the 5P model, which is predictive, preventive, personalized, participatory, and psycho-cognitive, as a diagnostic and normative framework to assess national cancer prevention infrastructures. Focusing on Sweden, Germany, Romania, and the United Kingdom, we conducted a qualitative comparative analysis informed by structured policy mapping and literature review. Our findings revealed significant divergence in system alignment with 5P principles, particularly in participatory governance, personalization, and anticipatory planning. While high-income countries tend to have better predictive infrastructure and screening coverage, they often lack formal mechanisms for community engagement and tailored communication. In contrast, under-resourced systems show lower alignment across multiple domains, underscoring the risks of top-down, technocratic strategies without trust-building or contextual sensitivity. We argue that the psycho-cognitive dimension, that is capturing values, perceptions, and behavioral drivers, is essential to translating prevention into practice. We conclude with actionable policy recommendations, emphasizing participatory infrastructure, cross-sectoral foresight, and ethical evaluation as core components of sustainable cancer control.

## Introduction

Cancer remains a leading cause of morbidity and mortality in Europe, and its burden is expected to rise with population aging and continued exposure to modifiable risks. The Global Burden of Disease 2019 study attributed over 44% of global cancer deaths to preventable factors, mainly tobacco, alcohol, poor diet, and physical inactivity [1, 2]. Although metabolic risks such as obesity and high fasting glucose are rising in importance, behavioral determinants still account for the majority of avoidable cancer cases.

In 2021 alone, over 1.1 million deaths in the EU, more than 21% of the total, were attributed to preventable risk factors [3]. These burdens are deeply unequal: adolescent obesity rates are 60% higher in low-income groups, and life expectancy varies by up to eight years across member states [3]. Aging adds urgency to this challenge. By 2050, nearly one-third of EU residents will be over 65, with over 40% expected to live with multiple, largely preventable chronic conditions [3]. The causal relationship between cancer risk and lifestyle has received growing attention in recent years [4–6]. Evidence supports the effectiveness of both lifestyle-based indices (such as Healthy Lifestyle Index Score), and precision prevention strategies [7–13] that reduce incidence through targeted behavioral and early detection interventions [14]. Despite this knowledge base, many European health systems continue to allocate resources primarily to curative care [15]. This stands in stark contrast to the historical record, in which public health interventions such as sanitation, vaccination, and maternal-child health were central drivers of gains in life expectancy over the twentieth century [16–18].

This paper takes the position that primary prevention needs to be the foundation of future cancer control. Although preventive strategies are well-documented and technically feasible, they remain chronically underprioritized, both in funding and policy design. We argue that one of the reasons for this is a mismatch between how prevention is implemented and how people understand, trust, and act on preventive messages. To address this, we emphasize a critical, often neglected component of prevention systems, the psycho-cognitive dimension. Within the 5P framework, that is predictive, preventive, personalized, participatory, and psycho-cognitive [19], the psycho-cognitive component is often described as the 5th P. This dimension highlights motivation, perception, cultural framing, and trust as determinants that may influence whether preventive interventions are effective in real-world contexts. While originally developed within clinical medicine, the 5P model has evolved into a broader framework for anticipatory, person-centred public health. Its core value lies in aligning prevention systems not only with data and technology, but with human behavior, equity, and emotional context.

Using examples of primary prevention (HPV vaccination), and secondary prevention (cervical screening), we assessed how four health systems, Sweden, Germany, Romania, and the United Kingdom, align with the 5P model. We focused on HPV vaccination and cervical cancer screening as archetypes of primary and secondary prevention because they are underpinned by strong evidence, are prioritised in international cancer-control strategies, and are measured consistently across countries. HPV vaccination prevents acquisition of oncogenic HPV types thereby reducing cervical cancer incidence at the population level while cervical screening (cytology and HPV testing with follow-up) detects and treats pre-cancerous lesions, preventing progression to invasive disease [20]. Both programmes have clear coverage indicators, robust registries, and well-defined delivery pathways, which makes them suitable for cross-country comparison and for operationalising the 5P framework.

The paper then suggests that prevention depends not only on infrastructure but also on behavioral alignment and emotionally informed practice. We conclude with recommendations to incorporate psycho-cognitive and participatory design approaches into national cancer strategies and to treat prevention as a central moral, clinical, and economic priority for sustainable health systems.

## Conceptual framework: 5P in cancer prevention

Cancer prevention offers a distinctive opportunity for health-system innovation because its burden is quantifiable, its major risk pathways are well characterized, and multiple evidence-based interventions exist across behavioral, clinical, and population-level (structural and policy) domains [1, 21, 22]. In theory, prevention represents a high-yield investment for health systems because interventions like vaccination, taxation, and screening can reduce long-term burdens on both individuals and institutions [23]. In practice, however, policy inertia, commercial resistance, and weak public engagement have limited progress. Even when effective tools exist, such as smoke-free legislation or alcohol pricing, they often face political pushback and suffer from unstable funding [24]. Public health campaigns also struggle for visibility, with anti-smoking messages frequently outspent by commercial advertising at ratios as high as 1:100 [25]. Available tools are not consistently translated into sustained, equitable implementation.

To address these challenges, we propose the 5P framework as an organizing conceptual framework for system-level cancer control, linking prevention, early detection, and care delivery across governance and implementation [19]. Extending on the concept of P4 medicine, predictive, preventive, personalized, participatory [26], by adding a critical fifth dimension, the psycho-cognitive aspect, Gorini and Pravettoni argue that even personalized medicine must do more than tailor interventions biologically. It must account for individual psychological needs, cognitive styles, decision-making preferences, and emotional responses to illness and care. They advocate for a patient-centered model where individuals are empowered through clear, personalized information, improved informed consent processes, shared decision-making, and interactive, tech-based support tools. They maintain that psychological alignment enhances quality of life, treatment adherence, and mental health outcomes such as reduced anxiety and depression, thereby reinforcing the efficacy of biomedical interventions. This psycho-cognitive engagement is particularly urgent for primary prevention, where the perceived benefits are often abstract, the barriers emotional, and the uptake socially influenced.

In other words, the 5P model indicates:

- *Predictive*: Use of epidemiological data, behavioral risk models, and registries to identify high-risk populations and allocate resources accordingly.
- *Preventive*: This pillar integrates primary (risk reduction), secondary (early detection), and tertiary (rehabilitation and survivorship care) prevention into a unified strategy.
  o Primary prevention- in the case of cancer- targets root causes such as tobacco, poor diet, alcohol, sedentary lifestyles, and air pollution, which account for about 40% of preventable cancer burden in Europe [27–29].
  o Secondary prevention, especially cancer screening, remains underutilized. Cervical screening coverage (the example used in this study) in several EU member states remains below 50%, often without effective follow-up [30, 31].
  o Tertiary prevention includes survivorship care and rehabilitation, which are better funded but offer lower returns if upstream interventions are neglected. Most health systems still direct the bulk of resources toward tertiary care [32].
- *Personalized*: Tailor outreach and service delivery to demographic, cultural, and regional diversity. A personalized approach to prevention can be understood as risk-stratified public health (often discussed under ‘precision public health’), in which prevention strategies are tailored to individuals or well-characterized population subgroups based on demographic, behavioral, clinical, and environmental risk profiles, with the aim of delivering the right intervention to the right group at the right time [33–35]. This form of personalization does not require genomic data but rather leverages stratified public health design to improve equity and effectiveness in upstream interventions [9, 11, 36].
- *Participatory*: Meaningful community involvement in the co-design, implementation, and evaluation of prevention programmes can strengthen perceived legitimacy and trust, and is frequently associated with improved acceptability and uptake [37]. Top-down campaigns often fail without local engagement.
- *Psycho-cognitive*: Psychological and emotional factors such as fear of diagnosis, stigma, health literacy, or mistrust can undermine prevention, even when services are free and available [13, 19]. Designing strategies that account for patient autonomy, communication needs, and comprehension barriers is therefore essential.

The 5P model thus reframes cancer prevention as not just a technical or clinical challenge, but also a human-centered process, one that demands alignment between institutional design, structural incentives, and citizens’ cognitive and emotional realities.

## Data and Methods

This paper adopts a qualitative comparative case study approach to analyze national cancer prevention strategies across four European countries: Sweden, Germany, Romania, and the United Kingdom. These cases were selected to reflect variation in structural capacity, governance models, and implementation maturity of prevention systems. Countries were selected using three predefined criteria: investment level in public health and prevention (as % of GDP and health spending- as shown in Table 1); governance structure (centralized, decentralized, or federal); implementation maturity, assessed via HPV vaccination coverage, cervical cancer screening rates, and continuity of national cancer prevention strategies.

**Table 1:** Case selection matrix for comparative analysis

Country	Expenditure on preventive healthcare, 2022 Relative to GDP	Governance model	Implementation maturity^1^	Implementation notes	Rationale for inclusion/exclusion
Sweden	0.39%	Regional execution [43]	87% HPV 78% screening Swedish National Cancer Strategy	Adopted 2009, still active (regional RCC model); Longstanding strategy coordinated via six Regional Cancer Centres (RCCs); continuously updated	Selected: High-capacity system (benchmark case)
Germany	0.99%	Federal [44] (Länder-based health policy)	55% HPV 47% screening (women aged 20-34; cytology, 2022) National Cancer Plan (Nationaler Krebsplan)	Launched 2008; active Federal implementation, coordinated by BMG, DKFZ, and cancer societies; updated regularly	Selected: High spending, low coherence, federal fragmentation
Romania	0.16%	Centralized [45], under-resourced	17% HPV 6% screening Planul Național de Control al Cancerului 2023–2030	Adopted Jan 2022 Implementation in early stages; aligns with Europe’s Beating Cancer Plan	Selected: Illustrative of chronic underinvestment and policy gaps
United Kingdom	0.9%	Centralized [46](England NHS-led)	73.5% HPV 74.9% Screening NHS England Cancer Delivery Plan 2023–2025	Updated 2023 Covers England; addresses prevention, screening, and outcomes; legacy continuity from earlier plans	Selected: Legacy capacity but capacity constraints following prolonged fiscal pressure
France	0.48%	Centralized	45% HPV 54% screening 10-Year Strategy to Fight Cancer (2021–2030)	Active since 2021 Successor to previous three National Cancer Plans; aligned with EU targets	Not selected: Profile overlaps with UK, less contrast offered
Italy	0.54%	Decentralized (regional delivery)	51% HPV 41% screening National Prevention Plan (PNP) 2020–2025 + cancer-specific objectives	In effect 2021–2025 Coordinated by Ministry of Health and Regions; delivery varies by region	Not selected: Governance complexity overlaps with Germany
Poland	0.12%	Centralized	13% HPV 11% screening National Oncology Strategy (Strategia Walki z Rakiem)	2020–2030 Adopted in 2020; criticized for limited early implementation	Not selected: Similar profile to Romania, fewer data sources
Netherlands	0.58%	Centralized	63% HPV 49% screening National Cancer Control Plan (KWF-led + RIVM public health integration)	Ongoing since 2020s Integrated in general public health and prevention frameworks; high registry use	Not selected: Very similar to Sweden, less contrast
Hungary	0.19%	Centralized	75% HPV 34% screening National Cancer Control Programme	Updated version active in 2020s Exists but not detailed or strongly evaluated; low CAYA focus	Not selected: Data availability issues, similar to Romania
Finland	0.62%	Centralized	62% HPV 72% screening Finnish Cancer Control Strategy (FICAN-led)	Renewed 2020–2024 Strong coordination via Finnish Cancer Society and National Institute for Health and Welfare	Not selected: Nordic model already covered by Sweden; added for contrast

^1^Data on HPV vaccination were derived from [39] (female coverage, latest available 2024 data where available). Cervical screening participation data were derived from OECD / EU Country Cancer Profile sources [41, 42] and reflect the latest available country-level estimates; figures are not fully harmonised across countries in year, denominator, age group, or source type. For Germany, the reported 47% refers to the 2022 cytology screening rate among women aged 20–34; programme-wide participation data for the full eligible population were not available [41]. Investment data were derived from [47]; see also [48]. Type of health system governance (centralized, federal, or decentralized) was classified using WHO / European Observatory reports [43–46]. Implementation maturity was assessed through: (a) national female HPV vaccination coverage; (b) cervical screening participation; and (c) institutional continuity (e.g. national cancer plans in place during 2021–2024)

*GDP*. gross domestic product; *HPV*. human papillomavirus; *RCC*. regional cancer centre(s); *BMG*. Federal Ministry of Health (Bundesministerium für Gesundheit, Germany); *DKFZ*. German Cancer Research Center (Deutsches Krebsforschungszentrum); *NHS*. National Health Service; *EU*. European Union; *KWF*. Dutch Cancer Society (KWF Kankerbestrijding); *RIVM*. National Institute for Public Health and the Environment (Rijksinstituut voor Volksgezondheid en Milieu, Netherlands); *CAYA*. Children, Adolescents and Young Adults; *FICAN*. Finnish Cancer Center

This selection process was informed by publicly available data from OECD Health Statistics [3, 38], WHO Europe reports [39], and EU-level cancer control documentation [40]. A shortlist of ten countries was initially reviewed, and the final four were selected to maximize institutional and policy contrast across these dimensions. Table 1 presents the selection matrix, with investment, governance, and implementation variables summarized alongside rationale for inclusion or exclusion.

We conducted structured searches of peer-reviewed literature using PubMed, Scopus, and Google Scholar (2015–2024), using country-specific Boolean combinations related to “cancer prevention,” “public health systems,” “screening,” “HPV vaccination,” and “system reform.” Searches covered 2015–2024; comparative extraction focused on 2021–2024 to represent current policy and implementation status, with earlier sources retained selectively for context.

National policy and grey literature were identified via hand-searching the official websites of national health ministries and public health agencies, and through EU-level portals such as the European Cancer Organisation [40], WHO Europe [39], and OECD Health Observatory [32, 43–45 and 49, 50].

Inclusion criteria for documents were: (1) relevance to national cancer prevention policies or infrastructure; (2) date of publication between 2021 and 2024; (3) focus on system-level or institutional strategies; and (4) source credibility, defined as peer-reviewed, government-issued, or from recognized international institutions.

Each P domain is interpreted as a systems-level function that contributes to effective and equitable prevention:

- *Predictive*: Use of epidemiological modeling, registries, and behavioral surveillance to forecast risk and guide strategy.
- *Preventive*: Deployment of upstream and midstream interventions (HPV vaccination, screening infrastructure, and environmental risk reduction).
- *Personalized*: Tailoring of outreach and delivery by demographic or behavioral segmentation (culturally adapted HPV programmes) [9, 36].
- *Participatory*: Civic engagement in policy design, implementation, and evaluation (stakeholder forums, co-production).
- *Psycho-cognitive*: Alignment of communication and service design with population beliefs, trust, motivation, and emotional literacy. The psycho-cognitive domain is central to our analysis, as it often determines whether preventive tools are used, understood, or rejected. For example, low HPV uptake may reflect not logistical failure but fear, mistrust, or perceived stigma [8, 19]. We used the OECD Patient-Reported Indicator Surveys (PaRIS) primary-care survey of people aged ≥ 45 living with one or more chronic conditions (2023–24 wave [52, 53]). PaRIS reports standardised patient-reported indicators grouped into domains covering (i) experience of care e.g., person-centred care and care coordination and (ii) empowerment e.g., trust, support to self-manage, and digital health literacy. For the 5th P, we selected PaRIS indicators a priori as the closest system-level measures of empowerment and people-centredness in prevention. Countries not in PaRIS (Sweden, Germany) were assessed using national patient-reported experience measures or patient reported outcome measures (PREMs/PROMs) infrastructures and policy documents. It should be noted that PaRIS is not a cancer-specific dataset; it is a broader people-centred primary care / chronic care experience instrument, therefore PaRIS indicators are used here as contextual proxies for empowerment, trust, care co-ordination, and health literacy in the surrounding health system, not as direct measures of cancer prevention experience.

Qualitative evidence was extracted for each selected country and mapped to the 5P domains. Scoring was guided by the rubric shown in Table 2, with definitions and indicators per domain. Descriptive findings were then qualitatively coded and scored [1–4] for each P domain to reflect relative alignment. Scoring was based on observed patterns of policy consistency, institutional continuity, infrastructure development, and community engagement.

**Table 2:** Scoring rubric for 5P alignment assessment

Score	Definition	Indicators Considered
1	No evident alignment with the domain	Absence of national policy, registries, preventive budget, or targeted programmes
2	Limited or fragmented alignment	Inconsistent implementation; localized or pilot initiatives; poor uptake or lack of continuity
3	Moderate or emerging alignment	National strategy exists but unevenly implemented; moderate coverage; gaps in participation/infrastructure
4	Strong and integrated alignment	Comprehensive policies with implementation across sectors; high coverage; continuity; stakeholder trust

PaRIS indicators were reported as percentages in the OECD release [53]. We recoded these into the 1-4 alignment categories shown in Table 2 using author-defined fixed cut-offs, selected for policy interpretability and to approximate quartiles of the OECD country distribution.

- *Person-centred care*: < 70% = 1, 70–79% = 2, 80–89% = 3, ≥ 90% = 4
- *Care coordination (experience)*: < 40% = 1, 40–59% = 2, 60–74% = 3, ≥ 75% = 4
- *Trust and support to self-manage*: < 50% = 1, 50–59% = 2, 60–69% = 3, ≥ 70% = 4
- *Digital health literacy*: ≤ 10% = 1, 11–19% = 2, 20–27% = 3, ≥ 28% = 4

For each country we combined the two psycho-cognitive sub-domains, Experience (person-centred care, care coordination) and Empowerment (trust and support to self-manage, digital health literacy), using a conservative minimum rule: the overall psycho-cognitive category is the lower of the two sub-domain categories. This reflects a conceptual maturity logic in which a shortfall in any necessary pillar limits overall alignment.

This scoring enabled us to identify which domains are most and least developed across health systems, and how the psycho-cognitive dimension may explain otherwise paradoxical outcomes, such as low uptake in well-funded systems or persistent inequities despite stratification tools.

## Results

The comparative findings are organized to show why each score was assigned and how it relates to system context. We first situate the cases using OECD health-expenditure profiles (Table 3). Scores follow the Table 2 rubric and represent ordinal categories of alignment (from 1 = nascent to 4 = strong and integrated). To contextualise performance, Table 3 (adapted from [38]) summarises total and preventive health-expenditure profiles for each country as percentage of GDP and public health budget over 2021–2023.

**Table 3:** Expenditure and financing

Time period	2021	2022	2023
Reference area			
*Health function: Total as percentage of GDP*			
Germany	12.7	12.4	11.7
Sweden	11.3	10.9	11.3
United Kingdom	12.1	11.1	11.0
Romania	6.5	5.8	5.8
*Health function: Preventive care as % of GDP*			
Germany	0.9	1.0	0.6
Sweden	0.5	0.4	0.4
United Kingdom	1.5	0.9	0.6
Romania	0.2	0.2	0.1
*Health function: Information, education and counseling programmes as % of GDP*			
Germany	0.0	0.0	0.0
Sweden	0.1	0.1	0.1
United Kingdom	0.1	0.1	0.1
Romania			0.0
*Health function: Immunisation programmes as % of GDP*			
Germany	0.3	0.3	0.1
Sweden	0.1	0.1	0.1
United Kingdom	0.4	0.2	0.1
Romania	0.1	0.1	0.0
*Health function: Early disease detection programmes as % of GDP*			
Germany	0.3	0.4	0.1
United Kingdom	0.5	0.2	0.0
Romania	0.0	0.0	0.0
*Health function: Healthy condition monitoring programmes as % of GDP*			
Germany	0.1	0.1	0.1
Sweden	0.1	0.1	0.1
United Kingdom	0.2	0.3	0.3
Romania	0.0	0.0	0.0
*Health function: Epidemiological surveillance and risk and disease control programmes as % of GDP*			
Germany	0.2	0.2	0.2
Sweden	0.1	0.0	0.0
United Kingdom	0.2	0.1	0.1
Romania	0.1	0.1	0.0
*Health function: Governance and health system and financing administration as % of GDP*			
Germany	0.5	0.5	0.5
Sweden	0.1	0.1	0.1
United Kingdom	0.2	0.2	0.2
Romania	0.2	0.2	0.2

*Data source* [38]

**Fig. 1:**
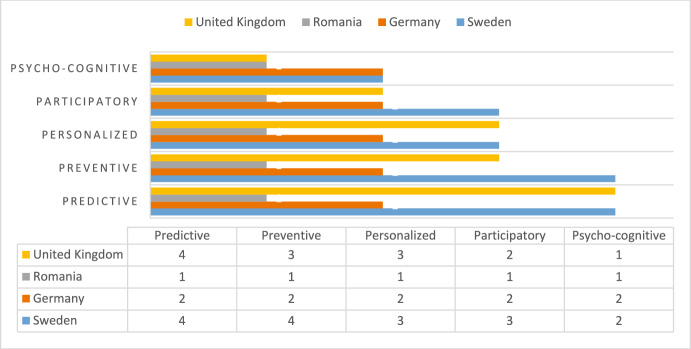
Alignment of national cancer prevention strategies with the 5P public health framework. Bars show ordinal alignment categories (1: nascent; 2: emerging; 3: established; 4: advanced) across five dimensions-predictive, preventive, personalized, participatory, and psycho-cognitive- for four European health systems. Scores were derived from triangulated evidence (policy documents, implementation reports, routine indicators) using the Table 2 rubric; they represent qualitative alignment categories and are not rankings. For the psycho-cognitive dimension we combined Experience (person-centred care, care coordination) and Empowerment (trust, support to self-manage, digital health literacy) using a bottleneck rule (final score: lower of the two sub-domains). PaRIS patient-reported indicators (2023–24) were used where available (Romania; Wales within the United Kingdom) and mapped to 1–4 using the cut-offs specified in Methods; Sweden and Germany were assessed qualitatively using national PREMs/PROMs and policy documentation. *PaRIS*, OECD Patient-Reported Indicator Surveys; *PROMs*, patient-reported outcome measures; *PREMs*, patient-reported experience measures; *PPI*, patient and public involvement; *FIT*, faecal immunochemical test; *HPV*, human papillomavirus; *SMS*, short message service (text) reminders; *SDM*, shared decision-making. Computation summary: Predictive, mean of three indicators (registry integration, risk-stratification in programmes, data linkage/timeliness); Preventive, HPV and screening coverage mapped to 1–4 cut-offs; Personalized, count of national features (self-sampling, SMS reminders, risk-stratified outreach, modality choice/FIT-first); Participatory, count of mechanisms (legal PPI, patient representatives on national committees, routine national PREMs); Psycho-cognitive, bottleneck of Experience and Empowerment as defined above

We then provide an integrated matrix that links each country’s predictive, preventive, personalized, participatory, and psycho-cognitive justifications to available evidence (Table 4). Figure 1 further provides a visual map of the domains.

**Table 4:** Integrated country profile and 5P justification table

Country	Predictive Justification	Preventive Justification	Personalized Justification	Participatory Justification	Psycho-Cognitive Justification
Sweden	Registry infrastructure; RCC-supported analytics; risk stratification capacity	High programme performance; structured RCC implementation; iterative updates	Stratified outreach in development; early co-production activity	RCC-linked participatory structures and co-production initiative	No PaRIS 2023–24 data; PROMs/PREMs expanding in pathways; patient representation in RCC governance; strong national portals; variable decision-aid uptake; registry-linked transparency improving; psychosocial support available, uneven distribution
Germany	Cancer registries established; linkage still developing	Moderate coverage; variable follow-through; high spending with uneven implementation	Predominantly biomedical targeting	Formal patient involvement but limited routine participatory governance	No PaRIS 2023–24 data; PROMs/PREMs not routinely embedded in prevention; formal involvement provisions, variable implementation; mixed health literacy/SDM support; limited linkage to commissioning; psychosocial support variable
Romania	Limited surveillance capacity; constrained anticipatory/risk tools	Very low coverage; under-resourced implementation capacity; early-stage national plan delivery	Limited tailored outreach; reliance on general national messaging	Top-down communication emphasis; low institutionalisation of civic engagement	PaRIS 2023–24: care co-ordination 78%; person-centred care 92%; self-management confidence 42%; trust 52%; digital health literacy 7%; general health 43%; physical health 52%; pattern: stronger experience, weaker empowerment/literacy
United Kingdom	NHS performance dashboards; registry integration; cross-nation variation	Strong HPV and screening performance; continuity from NHS frameworks	Self-sampling initiatives; SMS reminders; FIT-first pathways	Participation legacy; co-design capacity constrained by service pressures	PaRIS coverage: Wales only; below-average overall outcomes/experiences; support to self-manage 43%; trust 46%; care co-ordination experience 22%; strengths: digital health literacy 34%; practice preparedness 83%; electronic exchange 94%

The OECD PaRIS first wave covered 19 systems in 2023–24, including Romania and Wales (United Kingdom) [52]; Sweden and Germany did not participate in this wave. Psycho-cognitive indicators partly derive from broader person-centred health-system evidence (for example PaRIS), not solely cancer-specific measures; interpret as contextual proxies.

*PaRIS*: OECD Patient-Reported Indicator Surveys; *PROM*: patient-reported outcome measures; *PREMs*: patient-reported experience measures; *RCC*: Regional Cancer Centre; *PPI*: patient and public involvement; *FIT*: faecal immunochemical test; *HPV*: human papillomavirus; *SMS*: short message service (text) reminders; *SDM*: shared decision-making; *NHS*: National Health Service

### Sweden

Sweden allocates about 0.5% of its health budget to prevention and maintains high coverage in both HPV vaccination (87%) and cervical screening (78%) [3, 38]. However, Sweden’s personalized and participatory strategies remain limited in reach. National surveys show that immigrant groups and low-income populations continue to experience lower health literacy [54, 55], reduced screening participation [56], and limited engagement in preventive behaviors [57]. Rising obesity rates across socio-economic strata [58] highlight the need for prevention approaches that move beyond information provision to address motivational, cultural, and emotional factors [59]. At the same time, Sweden has taken significant institutional steps toward embedding person-centred care and shared decision-making into its healthcare system. National regulations, quality registries, and emerging learning health systems are being designed to support co-produced care, where patients and their families are active partners rather than passive recipients [60]. The joint implementation of shared decision-making, person-centred care and co-production represents an ambitious paradigm shift. However, these frameworks have not yet fully closed the gap in psycho-cognitive framing particularly among structurally disadvantaged groups. As such, while Sweden demonstrates strong commitment to participatory governance in principle [61], the practical realization of equitable and emotionally attuned prevention remains uneven, reinforcing the importance of tailoring strategies to motivational and cultural contexts.

### Germany

Germany spends about 13% of GDP on health, the highest in the EU, but allocates only a small fraction to prevention, about one percent (which is still among the highest shares in the EU) [38, 62]. In 2023, less than 0.5% of health spending went to screening, and HPV vaccination coverage remained uneven, 55% for girls and just 27% for boys [63]. The federal structure leads to fragmented data systems and inconsistent prevention efforts across states. Efforts such as the Prevention Act (Präventionsgesetz 2015) signal a turn toward prediction and proactivity, yet implementation has been patchy. Importantly, prevention in Germany remains heavily individualized, with limited structural engagement with communities, however this could also be interpreted as the aftermath of COVID-19 and its effect on resource-allocation [64]. Finally, psycho-cognitive determinants (trust, cultural framing, and affective barriers) appear less consistently integrated into prevention communication and delivery than biomedical and service-availability considerations [65]. For a comprehensive study on the state of prevention in Germany see Zeeb et al. [62].

### Romania

Romania records an HPV incidence 2.5 times the European average and a mortality rate more than four times higher [66]. The country invests less than 3% of its health budget in public health [3]. HPV vaccination rates remain very low (about 17%), and screening uptake is just 6% [67]. Predictive infrastructure is weak, and outreach programmes often fail to connect with rural populations, marginalized groups, and communities with low health literacy. Early HPV vaccine rollouts was discontinued due to low trust and weak stakeholder engagement, underscoring the limits of top-down strategies without participatory infrastructure [68]. The ongoing ReThinkHPVaccination project seeks to rebuild HPV vaccination strategies in Romania (and other European states) [66]. The collapse of Romania’s initial HPV vaccination campaign underscores the critical importance of psycho-cognitive design in public health. Mistrust, parental fears, lack of transparent informed consent processes, and top-down communication strategies contributed to widespread public resistance [69]. While the ReThinkHPVaccination initiative aims to rebuild these relationships through improved outreach and system reform, infrastructure alone will not be sufficient. PaRIS report also indicates strong interpersonal experience (person-centred care 92%) and co-ordination (78%) alongside low empowerment (confidence to self-manage 42%) and low trust (52%), with digital health literacy among the lowest (7%).

### United Kingdom

The United Kingdom has historically emphasised prevention through the NHS and public-health agencies (including Public Health England, with functions subsequently redistributed across successor organisations from 2021) [70, 71]. About 4% of the health budget goes to public health [72], supporting high HPV vaccination coverage (dose 2 coverage in year 10 females was 73.5% [73]) and strong cervical screening uptake (74.9% for women aged 50 to 64, and 67.5 for women aged 25–49 [74]). Predictive capacity rests on comprehensive cancer registries and risk modeling [75]. Since 2010, austerity has weakened local governance, reducing community participation and proactive planning [76, 77]. Adaptive screening and behavioral analytics continue, but long-term sustainability remains uncertain without renewed structural investment [70].

We observed divergent psycho-cognitive profiles across participating systems. Romania combines very high person-centred care (92%) and care co-ordination (78%) with low confidence to self-manage (42%), low trust (52%), and very low digital health literacy (7%) among people with chronic conditions. In the United Kingdom, PaRIS data are available for Wales only; Wales reports below-average patient outcomes and experiences overall, with support to self-manage 43%, trust 46%, and co-ordination experience 22%, despite strong digital health literacy (34%), practice readiness to co-ordinate (83%), and electronic record exchange (94%). Sweden and Germany did not participate in the 2023–24 PaRIS wave; for these countries, psycho-cognitive assessment draws on national patient-experience infrastructures (e.g., Sweden’s Nationell Patientenkät) and policy documentation rather than PaRIS.

## Discussion

From this analysis, three insights emerge. First, alignment with the 5P model does not map straightforwardly onto GDP or aggregate health expenditure, instead, governance quality, policy coherence, and institutional maturity appear to mediate how resources translate into preventive capacity. Second, predictive capacity and psycho-cognitive alignment were comparatively underdeveloped in our cross-case assessment. Even where digital tools and analytics exist, limited interoperability, uneven integration of behavioural and implementation science, and variable user-centred design constrain anticipatory governance and adaptive delivery. Third, participation and personalisation are consequential but unevenly institutionalised, namely that lower-resourced systems often face capacity constraints for tailoring and co-creation, while highly technocratic systems may encounter challenges in generating relational trust and cultural fit.

Consistent with prior studies, we observed that total health spending does not predict stronger prevention systems. Germany, for instance, shows weaker performance on prevention despite high expenditure, a pattern also noted in WHO Europe’s Health Systems Response Monitor and national assessments [62]. This discrepancy is not solely a reflection of federal fragmentation, but of broader institutional and political-economy factors that shape prevention priority-setting, governance, and implementation. Zeeb et al. [62] attribute this in part to the marginal role of prevention in political and medical cultures shaped by a historically dominant biomedical paradigm. They maintain that possible contributing factors include limited political prioritisation of prevention, competing commercial interests, and difficulties translating public health evidence into policy and practice. Delays or uneven progress in upstream measures, such as sugar-sweetened beverage taxation, tobacco and alcohol control, and restrictions on the marketing of unhealthy foods, are consistent with these broader implementation challenges [62].

Beyond financing and governance, predictive and psycho-cognitive capacities remain underdeveloped. These require interoperable surveillance, behavioral-science integration, and communicative strategies tailored to diverse populations. The absence of value-sensitive engagement and culturally responsive communication undermines both uptake and equity in preventive services, even when technically available.

Disparities in prevention are mirrored in research priorities. According to Schmutz et al. [78], primary prevention remains the least supported area in cancer research across Europe. Less than 4% of the 1,477 identified European cancer research funders show meaningful interest in primary prevention, while more than half prioritize secondary prevention. Schmutz et al. further note that not-for-profit organizations make up 35% of European cancer prevention research funders, while representing only 8% of all not-for-profit organizations funding cancer research. This research-policy misalignment reinforces the systemic undervaluation of upstream interventions and highlights the urgent need for structural rebalancing in both funding and research agendas.

Our study identified additional structural asymmetries. While recent studies including the IHE Comparator Report [79] and analyses by Horgan et al. [80] and Ádány et al. [81] document cross-country variation in screening uptake, vaccination coverage, and institutional coordination, such analyses typically foreground quantitative outputs or disease-specific outcomes. By contrast, our model foregrounded systemic capacities particularly personalization, participation, and psycho-cognitive alignment as critical yet often overlooked determinants of prevention efficacy.

Recent studies further substantiated these findings. For instance, Ola et al. [82] documented inequities in colorectal cancer screening across Europe, with markedly lower uptake in countries lacking national screening programmes. Participation was consistently associated with structural and social factors such as education, rurality, and healthcare access. Similarly, Gudmundsdottir et al. [56] and Mona et al. [57] identifeid persistent disparities in cancer screening and health literacy among immigrant and socioeconomically disadvantaged populations. These findings point to the limitations of universal availability and highlight the need for culturally tailored, trust-oriented strategies that integrate behavioral science and patient engagement.

Our findings also complemented research by Denny et al. [28] and Braveman et al. [83] showing that structural and social inequalities underlie differential prevention outcomes. The psycho-cognitive dimension provides a conceptual bridge between personalized care and public health engagement. It draws attention to the psychological, emotional, and cognitive conditions under which individuals interpret risk, engage with services, and make health-related decisions. As such, it enables a more holistic understanding of why prevention programmes fail or succeed, particularly in diverse, multi-ethnic populations.

These findings are consistent with comparative analysis across Europe, including those from the European Cancer Information System (ECIS), the Joint Action on Cancer Control, and the European Cancer Inequalities Registry, all of which document persistent disparities in screening uptake, HPV vaccination, and mortality outcomes across member states.

## Limitations

This analysis has several limits. It covers four purposively selected countries; differences in data and governance reduce direct comparability; and qualitative synthesis cannot prove causation. The 5P psycho-cognitive model also needs further testing beyond cancer. Future work should link psycho-cognitive indicators (decisional conflict, literacy) to registry outcomes, run pragmatic trials of low-friction delivery at scale, and use distributional cost-effectiveness to judge both value and fairness.

While the psycho-cognitive dimension adds critical nuance to prevention system analysis, we recognize that its operational boundaries remain evolving. The concept intersects with existing behavioral health models- such as the Health Belief Model, Theory of Planned Behavior, and COM-B (Capability, Opportunity, Motivation-Behavior) but also aims to go beyond them. Unlike frameworks focused narrowly on behavior change capacity, the psycho-cognitive lens examined here emphasizes meaning-making, trust, emotional resonance, and cultural cognition as central to prevention engagement. Future research should more rigorously distinguish this dimension both conceptually and empirically, including how psycho-cognitive design standards interact with decision-making preferences, belief systems, and social norms in diverse populations. Mixed-methods or ethnographic work could further ground this concept in lived experience, improving both measurement and system responsiveness.

Additionally, our 5th P assessment uses system-level proxies (PROMs/PREMs, patient involvement, health-literacy and decision-support policies) rather than individual-level measures, consistent with WHO and OECD people-centred frameworks, country scores may therefore under or overestimate patient experience variability. In future studies we hope to be able to survey the psycho-cognitive dimension of 5P medicine emphasising patient empowerment, shared decision-making, and quality of life and operationalizing it with system-level indicators of people-centeredness (e.g., voice, choice, co-production, respectfulness, integration).

## Policy recommendations

Improving cancer prevention performance and equity in European health systems is unlikely to be achieved through incremental programme adjustments alone. Our comparative 5P assessment suggests that prevention depends on how systems convert resources into coherent delivery- through governance, data integration, participatory capability, and psycho-cognitive alignment- rather than on aggregate health expenditure alone. On this basis, we proposed a 5P-informed agenda in which the psycho-cognitive pillar (decision support, motivation, health literacy, and trust) is treated as a design requirement alongside predictive, preventive, personalised, and participatory functions.

### Predictive and accountable data integration

National linkage across cancer registries, immunisation systems, and screening datasets; routine reporting on timeliness and equity gaps; locally actionable dashboards. Where feasible, inclusion of a minimal set of psycho-cognitive implementation markers (prior non-attendance, preferred communication channel, and brief proxies for health literacy/decision support needs). These steps operationalise the predictive pillar and reflect the cross-case observation that stronger coherence and linkage capacity tend to align with more reliable follow-through than fragmented arrangements.

### Finance and deliver a core prevention package

A defined, multi-year prevention budget line to protect delivery of evidence-based measures (tobacco control, HPV vaccination, organised cervical screening, FIT-based colorectal screening pathways where appropriate, and relevant environmental/occupational risk protections), accompanied by transparent coverage and equity reporting. This recommendation reflects our finding that prevention performance appears more closely related to the structure and protection of financing prevention and delivery than to total health spending alone.

### Personalised, equity-oriented delivery

Risk- and context-stratified invitations; modality choice and self-sampling where appropriate; navigator or outreach support for groups with lower completion. Programme accountability tied to gap reduction (absolute and relative) by socioeconomic status and migration background. This recommendation follows the persistence of within-country inequities described in the country assessments and supports a shift from uniform offers to needs-weighted delivery.

### Participatory governance and trust infrastructure

Institutionalised co-design and feedback loops with patient groups and relevant community organisations (including youth/parent councils and migrant organisations where relevant), with locally tailored outreach delivered via trusted messengers and routine local reporting-back of results. This operationalises the participatory pillar and is consistent with our comparative observation that more developed participation mechanisms are more commonly present in higher-uptake contexts.

### Psycho-cognitive design standards

Service designs that minimise cognitive and logistical burden (e.g., pre-scheduled appointments with easy rescheduling options, mailed FIT with prepaid return, concise reminders via more than one channel, and clear decision support at point of care). These measures translate the psycho-cognitive pillar into implementable standards and directly address barriers highlighted in the psycho-cognitive analysis (trust, comprehension, and completion losses along the pathway).

Finally, we note that psycho-cognitive and participatory approaches require institutional capacity. In under-resourced settings, implementation may need to prioritise low-cost, scalable components (reminders, culturally adapted materials, and community-based outreach), recognising that effectiveness is contingent on local delivery infrastructure and baseline trust conditions.

## Conclusions

This study proposed the 5P model as an organising framework for comparing how health systems structure cancer prevention. The framework is intended to complement conventional indicators of coverage and outcomes by foregrounding upstream policy and financing choices, service delivery and system capacity, population reach, and the cognitive and behavioural conditions that may shape prevention uptake. Used as a structured checklist, the 5P framework may assist policymakers in reviewing prevention pathways, identifying plausible implementation constraints, and selecting interventions that are feasible within local resource and governance conditions. In particular, incorporating participatory governance and psycho-cognitive design considerations alongside more traditional system components may help improve acceptability and usability of prevention services. Future research could operationalise the five pillars as measurable indicators and examine their association with prevention uptake and equity across settings, including through designs that better support causal inference.

## Data Availability

All data used in this study are publicly available and cited within the manuscript.
